# The HopQ1 Effector’s Nucleoside Hydrolase-Like Domain Is Required for Bacterial Virulence in Arabidopsis and Tomato, but Not Host Recognition in Tobacco

**DOI:** 10.1371/journal.pone.0059684

**Published:** 2013-03-26

**Authors:** Wei Li, Yi-Hsuan Chiang, Gitta Coaker

**Affiliations:** Department of Plant Pathology, University of California Davis, Davis, California, United States of America; Virginia Tech, United States of America

## Abstract

Bacterial pathogens deliver multiple effector proteins into host cells to facilitate bacterial growth. HopQ1 is an effector from *Pseudomonas syringae* pv. *tomato* DC3000 that is conserved across multiple bacterial pathogens which infect plants. HopQ1’s central region possesses some homology to nucleoside hydrolases, but possesses an alternative aspartate motif not found in characterized enzymes. A structural model was generated for HopQ1 based on the *E. coli* RihB nucleoside hydrolase and the role of HopQ1’s potential catalytic residues for promoting bacterial virulence and recognition in *Nicotiana tabacum* was investigated. Transgenic Arabidopsis plants expressing HopQ1 exhibit enhanced disease susceptibility to DC3000. HopQ1 can also promote bacterial virulence on tomato when naturally delivered from DC3000. HopQ1’s nucleoside hydrolase-like domain alone is sufficient to promote bacterial virulence, and putative catalytic residues are required for virulence promotion during bacterial infection of tomato and in transgenic Arabidopsis lines. HopQ1 is recognized and elicits cell death when transiently expressed in *N. tabacum.* Residues required to promote bacterial virulence were dispensable for HopQ1’s cell death promoting activities in *N. tabacum.* Although HopQ1 has some homology to nucleoside hydrolases, we were unable to detect HopQ1 enzymatic activity or nucleoside binding capability using standard substrates. Thus, it is likely that HopQ1 promotes pathogen virulence by hydrolyzing alternative ribose-containing substrates *in planta.*

## Introduction

Plants are constantly exposed to diverse microorganisms, but disease is the exception rather than the rule. Plants possess a waxy cuticle on the outside of their leaves, thick cell walls, and preformed chemical barriers that deter the entry of multiple microorganisms. Plants also rely on their innate immune system to actively defend against pathogenic microbes. Plants use surface localized immune receptors to recognize conserved microbial features, such as bacterial flagellin [1]. Plants also rely on intracellular nucleotide-binding leucine rich-repeat (NLR) immune receptors to recognize pathogen effector proteins delivered inside host cells during infection [2]. A common output of NLR recognition is programmed cell death at the site of infection [2].

In order to cause disease and suppress host defense responses, gram negative bacterial pathogens deliver effector proteins into host cells via the Type Three Secretion System (TTSS). Plant pathogenic bacteria deliver a large number (20–40) of effectors into host cells during infection [3]. Collectively, effectors are essential for bacterial virulence [4]. A greater understanding of effector enzymatic activity and host targets has significantly impacted our understanding of plant biology and immune signaling. In the last several years, the enzymatic function of multiple effectors has been elucidated, primarily from *Pseudomonas syringae* pv. *tomato (Pto*), the causal agent of bacterial speck on tomato and Arabidopsis. For example, effectors can suppress immune responses by directly targeting immune receptors [5], [6], by interfering with downstream signaling processes [3], [7], or by inhibiting vesicle trafficking [8]. Many effectors act as eukaryotic enzymes to suppress host immune responses [9]. Effectors possessing cysteine protease (AvrRpt2 and AvrPphB), tyrosine phosphatase (HopAO1), E3 ligase (AvrPtoB), mono-ADP-ribosyltransferase (HopF2 and HopU1), and phosphothreonine lyase (HopAI1) activity have been biochemically characterized and implicated in suppressing plant innate immunity (reviewed in [9]).

Despite significant progress in understanding how pathogens target immune receptors and signaling proteins, their role in modulating host metabolism is less well understood. Pathogen infection has been shown to induce significant changes in the host metabolome. For example, virulent *Pto*, compared with a type III secretion mutant, was able to modify host small molecule profiles, significantly altering host metabolism within eight hours post-inoculation [10]. Purine, amino acid, and sugar metabolism are significantly altered during infection with virulent pathogens and may be key targets of effector proteins [10]–[12]. Recent work also demonstrates that perturbations in tryptophan amino acid homeostasis influence resistance to the obligate biotroph *Hyaloperonospora arabidopsidis* [13]. The Cmu1 effector from the fungal pathogen *Ustilago maydis* acts as a chorismate mutase that changes the host metabolic state through metabolic priming for pathogen benefit [14]. Cmu1 likely acts to increase the flow of chroismate from the plastid to the cytosol, resulting in decreased salicylic acid biosynthesis. These findings indicate that the host metabolic state significantly impacts pathogen growth and virulence. Thus, targeting key aspects of host metabolism may be an effective and conserved pathogen virulence strategy.

Here, we investigated the role of the *Pto* HopQ1 (also known as HopQ1-1) effector in tomato and Arabidopsis. HopQ1 induces cell death when expressed in *Nicotiana benthamiana* and contributes to differences in host range in *P. syringae* pathovars [15], [16]. HopQ1 also slightly enhances disease symptoms and bacterial virulence on bean when expressed from *P. syringae* pv. *tabaci* [16]. Here, we investigated the similarity between HopQ1 and nucleoside hydrolases. HopQ1’s central region possesses some homology to nucleoside hydrolases (NHs), and putative NH catalytic residues are necessary to promote bacterial virulence. These same residues are not required for inducing cell death in *Nicotiana*, but are required for promoting virulence in Arabidopsis and tomato. We were unable to detect nucleoside hydrolase activity for purified HopQ1. HopQ1 has significant alterations in its N-terminal aspartate motif compared to characterized NHs, which is considered a hallmark of known NHs. Thus, it is likely that HopQ1 acts on novel plant-derived substrates potentially with ribose moieties.

## Results

### HopQ1 Possesses some Homology to Characterized Nucleoside Hydrolases

**Figure 1:**
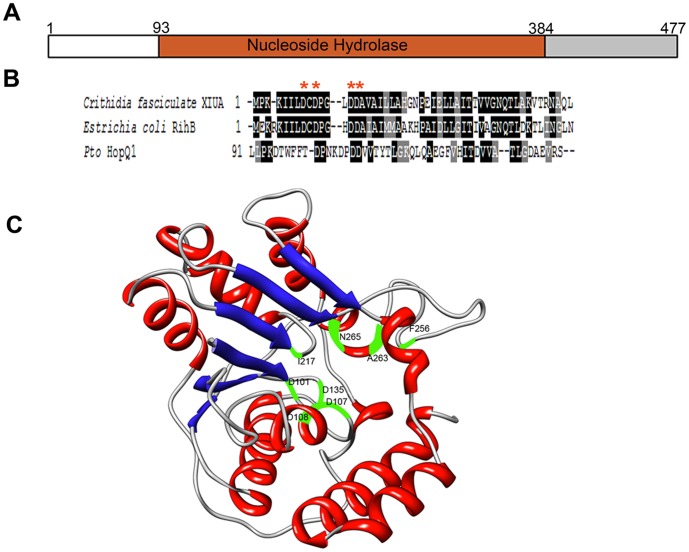
HopQ1 is a widely conserved effector with some homology to nucleoside hydrolases. (A) Domain organization of the *Pseudomonas syringae pv. tomato* DC3000 effector HopQ1. HopQ1’s N-terminal 62 amino acids possess a type III secretion signal; amino acids 93–384 possess homology to Nucleoside Hydrolases (NHs). HopQ1’s C-terminus does not possess homology to domains of known function. (B) ClustalW alignment of HopQ1 with the N-terminus of characterized IU-NHs. Key aspartate residues not conserved across HopQ1 and IU-NHs from *Crithidia fasciculata* (AAC47119) and *E. coli* RihB (A8A225) (C) A structural model of HopQ1_94–332_ using the *E. coli* IU-NH RihB (PDB ID 1Q8F) as a template. Key active site residues are highlighted in green, red = α helix, blue = β sheet.

HopQ1 is widely conserved across plant pathogenic bacteria. Homologs possess high sequence similarity across their effector domains (e-value  = 10^-145^, 98-49% amino acid identity) and can be identified in strains of *Pseudomonas, Xanthomonas, Ralstonia,* and *Acidovorax* as well as certain *Rhizobium* symbionts (Fig S1). HopQ1’s central region (amino acids 93–384) possesses significant homology to characterized nucleoside hydrolases (NHs, Fig. 1, Fig S1). NHs are enzymes that catalyze the cleavage of the N-glycosidic bond of a particular nucleoside, generating a ribose sugar and the respective base. A hallmark of NH activity is a recurring N-terminal aspartate DXDXXXDD motif [17]. Interestingly, HopQ1 and its homologs contain a variation on the aspartate motif, with DXXXDXDD in this position (Fig. 1B). This modification of the aspartate motif has not been previously identified in any *bona fide* NHs or other enzymes. Compared with characterized NHs from other organisms, HopQ1 and its homologs from phytopathogenic bacteria also have additional N- and C-terminal flanking sequences outside of their NH-like domain. HopQ1’s N-terminal 62 amino acids comprises the targeting signal for delivery via the type three secretion system into plant cells. HopQ1 also possesses a short linker (amino acids 63–92) and a C-terminal domain (amino acids 385–477) of unknown function (Fig. 1A).

In order to identify potentially important residues within HopQ1’s and to determine structural similarity between HopQ1 and known NH enzymes, the PHYRE protein fold recognition server was used to detect homology with known NHs [18]. All high-scoring matches were nucleoside hydrolases (e-value  = 10^-29^). Therefore, we generated a protein model of HopQ1 using the *E. coli* RihB (accession number U000007) NH as a template with the MODELLER software package [19] (Fig. 1C). A high confidence model was generated, indicating that HopQ1 protein may possess an NH-like fold (Fig. 1C).

### Putative HopQ1 Catalytic Residues are not Required for Eliciting Cell Death in *Nicotiana*

**Figure 2:**
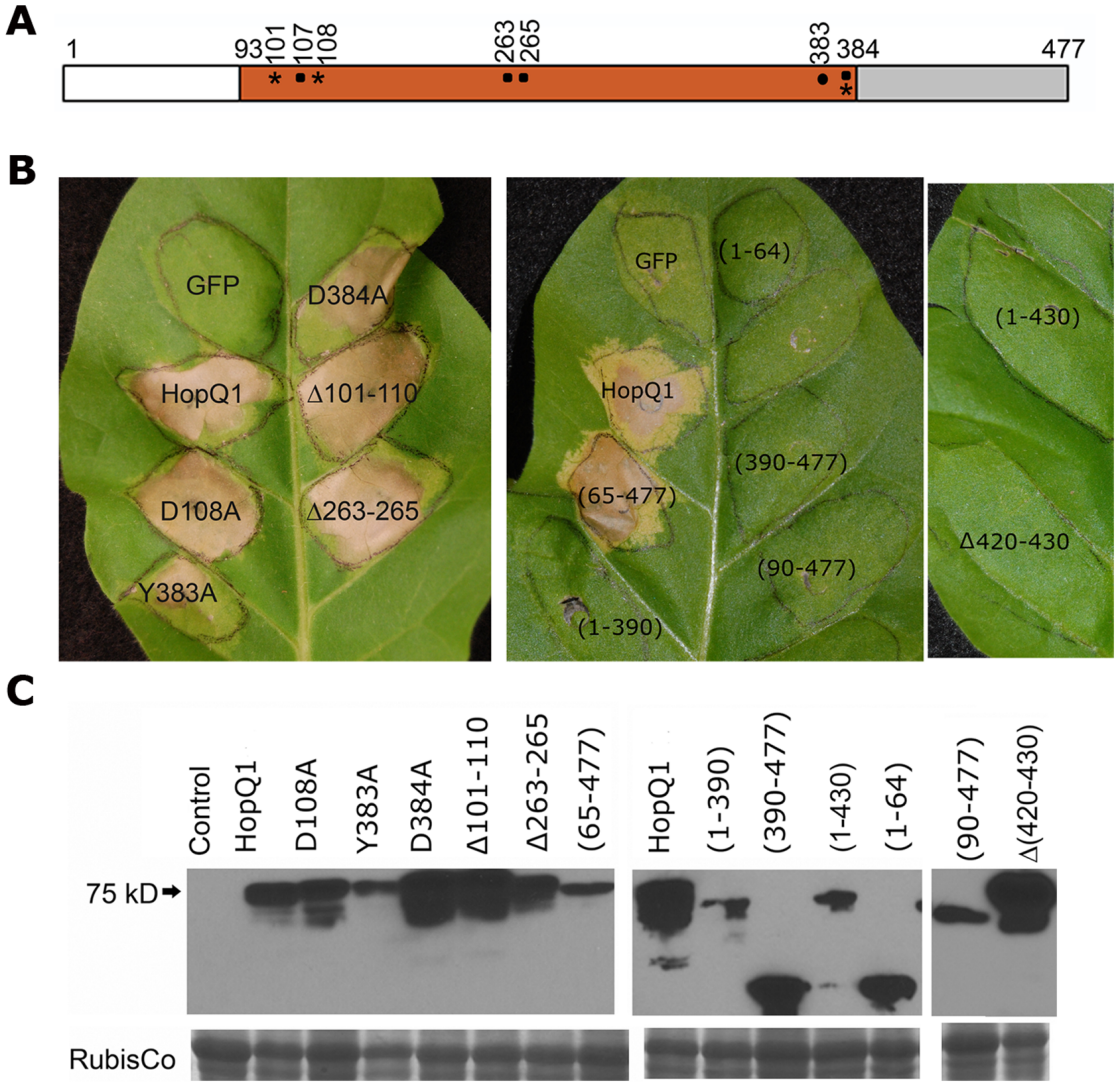
Structure-function of HopQ1 in *Nicotiana tabacum*. (A) Schematic representation of HopQ1 and corresponding mutation and truncations. Conserved putative NH catalytic residues involved in interactions with calcium ions, the ribose ring or substrate binding in characterized NHs are highlighted with an asterisk, square or dot on such positions, respectively. HopQ1 mutants were created by alanine substitution. (B) HopQ1 induces a hypersensitive response in *N. tabacum.* 35S::HopQ1:GFP was expressed in *N. tabacum* using *Agrobacterium-*mediated transient expression. Photographs were taken 72 h post-infiltration. Left panel: transient expression of HopQ1 and single or double deletions of conserved putative active site residues. Middle/right panels: Transient expression of HopQ1 truncations. (C) Western blots probed with anti-GFP showing expression levels of all constructs 40 h post-infiltration. Control = *N. tabacum* infiltrated with *Agrobacterium* expressing empty vector.

HopQ1 elicits cell death when expressed in *Nicotiana benthamiana* and deletion of *hopQ1* from *Pto* DC3000 enables this bacterium to cause disease on *N. benthamiana* [15]. Alternatively, expression of HopQ1_DC3000_ in *P. syringae* pv. *tabaci* (*Pta*), the causal agent of wildfire of tobacco, renders this pathogen unable to cause blight symptoms on *N. benthamiana* [15]. We found that *Agrobacterium*-mediated transient expression of HopQ1 in *N. tabacum* (tobacco) induces a robust cell death 48 h post-inoculation (Fig. 2).

The cell death phenotype in *N. tabacum* was used to dissect the importance of HopQ1’s NH-like domain in eliciting cell death. Conserved potential catalytic residues within HopQ1 were targeted for structure-function analyses. The residues selected for mutational or deletion analyses are involved in interacting with calcium ions (D101, D108, D384), the ribose ring (D107, A263, N265) and general substrate binding (Y383, D384) in well-characterized NHs [17], [20], [21]. HopQ1 and related mutations and deletions were transiently expressed in *N. tabacum* using *Agrobacterium-*mediated transient expression. The single amino acid substitution mutants HopQ1(D108A), HopQ1(Y383A) and HopQ1(D384A) were still able to elicit cell death (Fig. 2B, left panel). Truncations of key regions containing two (Δ263–265) or three (Δ101–110) putative catalytic residues also still elicited cell death (Fig. 2B, left panel). As a complementary approach, all HopQ1 constructs were expressed in *Pta* to test if they affected the ability to cause blight symptoms on *Nicotiana* (Fig S2, data not shown). The results between transient expression and pathogen inoculation were consistent; clones that could induce cell death rendered *Pta* unable to cause blight symptoms. Taken together, these data suggest that cell death induced by HopQ1 in *Nicotiana* does not require recognition of putative catalytic residues conserved in known NHs.

Outside of its NH-like domain, HopQ1 and its homologs have additional N- and C-terminal flanking sequences (Figs 1, S1). The N terminus is more variable compared to the rest of the protein sequence amongst different homologs, likely due to differences in type III secretion signals. Both the NH and C-terminal domains are highly conserved. Thus, contribution of individual domains within HopQ1 for eliciting an HR was investigated. Serial deletions within HopQ1 were created (Fig. 2). The N-terminus is not required for recognition when expressed in *N. tabacum,* as HopQ1_(65–477)_ still induced cell death (Fig. 2B, middle panel). Expression of HopQ1_(65–477)_ in *Pta* still enabled blight symptoms, likely because its TTSS is removed and the effector is not delivered into host cells (Fig S2B). Neither HopQ1_(90–390)_ nor HopQ1_(390–477)_ elicited cell death, suggesting the NH or C-terminal domain alone is not sufficient to induce recognition (Fig. 2B, middle panel). Multiple serial truncations of N and C-terminal pieces, an internal deletion of the NH, and deletion of a short internal region of the C-terminus were also generated. None of these deletion constructs were able to elicit cell death on *Nicotiana* (Fig. 2B, middle and right panels). These data indicate that only the N-terminus of HopQ1 is dispensable for recognition.

### HopQ1’s Nucleoside Hydrolase-like Domain Promotes Bacterial Virulence

**Figure 3:**
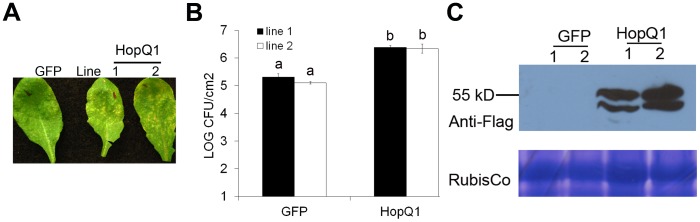
HopQ1 promotes bacterial virulence in transgenic Arabidopsis plants. Four-week-old plants expressing Dexamethasone (Dex) inducible HopQ1∶3XFLAG or GFP were sprayed with 30 µM Dex 24 h before syringe infiltration with 2x10^5^ cfu/ml of *P. syringae* pv. *tomato* DC3000. Four days post-inoculation, the plants were photographed (A) and subjected to growth curve analysis (B). Statistical differences were detected by Fisher’s LSD, alpha  = 0.01. Error bars represent means (n = 6) ±SE. (C) Anti-FLAG immunoblot illustrating protein expression in transgenic lines. Proteins were extracted from *Arabidopsis* leaves 24 h post-Dex treatment.

*Pto* DC3000 Δhopq1 does not exhibit a defect in pathogen virulence in Arabidopsis [15]. Therefore, to identify HopQ1 related disease phenotypes in Arabidopsis, we generated dexamethasone (Dex) inducible HopQ1∶3xFLAG lines. Inducing HopQ1 expression by spraying four-week-old plants with 30 µM Dex did not result in any obvious phenotypic differences in plant growth or health for up to 10 days post Dex application. When HopQ1 is expressed in plants, both full-length in addition to a slightly smaller cleaved version of the effector are detectable by western blot (Fig. 3C). Arabidopsis plants expressing HopQ1∶3xFLAG infected with *Pto* DC3000 exhibited ∼8–10-fold higher bacterial growth than controls, indicating that HopQ1 can act within plant cells to promote bacterial virulence (Fig. 3B).

**Figure 4:**
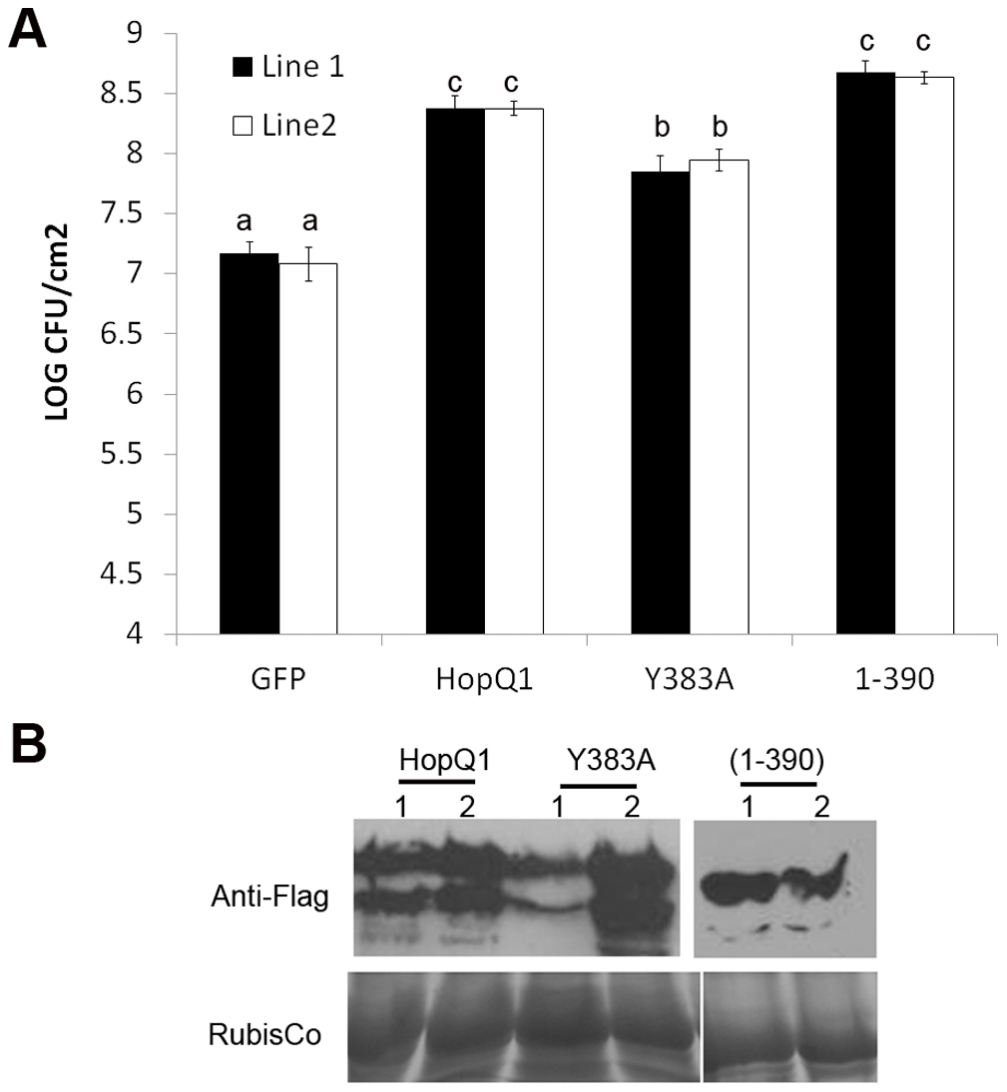
HopQ1’s nucleoside hydrolase-like domain is sufficient to promote bacterial virulence in transgenic Arabidopsis plants. (A) Four-week-old transgenic Arabidopsis lines expressing Dexamethasone (Dex) inducible HopQ1-3XFLAG, HopQ1_65–447_∶3XFLAG, and HopQ1_1–390_∶3XFLAG were subjected to bacterial inoculations. T3 homozygous lines were sprayed with 30 µM Dex 24 h before syringe-infiltration with 2x10^5^ cfu/ml of *P. syringae* pv. *tomato* DC3000. Four days post-inoculation, plants were subjected to growth curve analysis. Statistical differences were detected by Fisher’s LSD, alpha  = 0.01. Error bars represent means (n = 6) ±SE. (B) Western blots probed with an anti-FLAG illustrate HopQ1 expression in transgenic lines.

To determine which regions of the effector contribute to promoting bacterial virulence, homozygous T3 transgenic lines expressing HopQ1∶3xFLAG derivatives were also examined. Transgenic plants expressing either a C-terminal deletion (HopQ1_1–390_) still exhibited enhanced disease susceptibility phenotypes that were indistinguishable from wild-type HopQ1 lines (Fig. 4A). However, the putative catalytic mutant HopQ1(Y383A) exhibited an intermediate phenotype. These data indicate that the NH-like domain of HopQ1 can enhance bacterial virulence and the C-terminus is dispensable for this phenotype.

### HopQ1’s Nucleoside Hydrolase-like Domain is Required for Enhancing Bacterial Virulence when Delivered from DC3000

**Figure 5:**
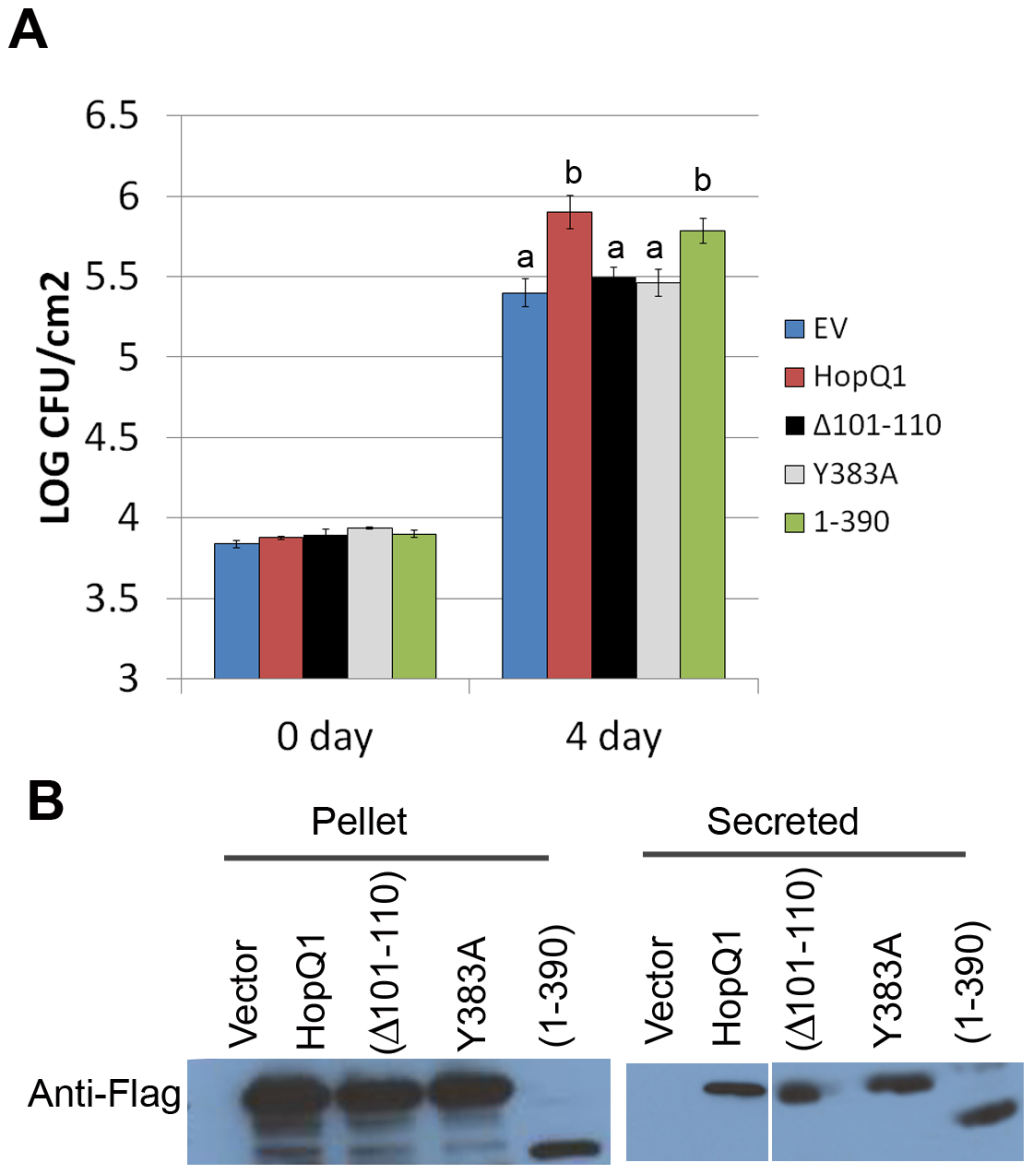
HopQ1’s putative catalytic residues are required for promoting bacterial virulence when delivered from *P. syringae* DC 3000 in tomato. (**A**) Expression of HopQ1 or HopQ1’s NH-like domain from the broad host range vector pBBR1 can complement the *Pto* DC3000 cluster IV deletion lacking the HopQ1, HopD1, and HopR1 effectors. Rio Grande 76R tomato plants were syringe infiltrated with 1x10^5^ cfu/mL of *Pto* DC3000 ΔIV expressing empty vector (EV), HopQ1∶3XFLAG, HopQ1(Δ101–110):3XFLAG, HopQ1(Y383A):3XFLAG, and HopQ1(1–390):3XFLAG. Growth curves illustrating bacterial population sizes are shown 0 and 4 days post-inoculation. Statistical differences were detected by Fisher’s LSD, alpha  = 0.05. Error bars represent means (n = 6) ±SE. The data shown are representative of three independent experiments with similar results. EV = empty vector. (**B**) *Pto* DC3000 ΔIV transformed with empty pBBR1 vector, or pBBR1 expressing HopQ1-3xFLAG expressing constructs were grown in hrp-inducing minimal media for 16 h at 18°C. The resulting bacterial pellet and precipitated secreted proteins were subjected to an anti-FLAG western blot to detect protein expression.

*Pto* DC3000 Δhopq1 exhibits reduced bacterial growth upon inoculation of the tomato cultivar Rio Grande 76R [22]. This defect in virulence can be complemented by adding expressing HopQ1 on a plasmid from either *Pto* DC3000 Δ*hopq1* or the cluster IV deletion (ΔIV) lacking the *hopQ1, hopD1,* and *hopR1* effectors [23]. The *Pto* DC3000 effectors are recognized in the Rio Grande 76R tomato line via the protein kinase PTO and NLR immune receptor PRF [24]. We investigated the role of HopQ1’s NH-like domain and putative catalytic residues by expressing them in *Pto* DC3000 ΔIV and inoculating these strains on Rio Grande 76R. We examined HopQ1(Δ101–110)_,_ HopQ1(Y383A), and HopQ1(1–390) after expression in *Pto* DC3000 ΔIV. Our results support the data we obtained with transgenic Arabidopsis plants. The HopQ1 putative catalytic mutant and deletion, Δ101–110 and Y383A, were unable to complement *Pto* DC3000 ΔIV (Fig. 5). However, HopQ1(1–390) was able to complement *Pto* DC3000 ΔIV (Fig. 5). All HopQ1 constructs possessed a C-terminal fusion to the 3XFLAG epitope. Using immunoblotting after induction in hrp inducing minimal media, we were able to see that all HopQ1 constructs were expressed in *Pto* DC3000 ΔIV and secreted via the TTSS (Fig. 5). Collectively, these results demonstrate that HopQ1’s NH-like domain is crucial for virulence promotion.

### HopQ1 Transgenic Lines Exhibit Altered Levels of Cellular Nucleosides

HopQ1 has some similarity to NHs and may affect plant nucleoside metabolism. To examine the effect of HopQ1 on plant nucleosides, nucleoside levels were quantified by LC/MS in Arabidopsis plants expressing Dexamethasone (Dex) inducible GFP or HopQ1∶3xFLAG. *Arabidopsis* plants were grown on ½ MS Agar supplemented with 30 µM Dex for three weeks before harvesting to quantify cellular nucleosides. The levels of the nucleosides uridine, cytidine, inosine, thymidine, adenosine and guanosine were measured (Fig S3). Transgenic plants expressing HopQ1∶3xFLAG exhibited significantly lower levels of uridine and cytidine (Fig S3A–B), in accordance with its similarity to IU-NHs. Levels of inosine, guanosine, and adenosine were not significantly different between HopQ1 and GFP expressing plants (Fig S3D–F). Surprisingly, we were able to detect higher levels of thymidine in HopQ1 expressing plants than in GFP expressing plants. Multiple aspects controlling nucleoside metabolism and salvage remain unclear in plants. As alterations in nucleoside metabolism can affect a wide variety of metabolic processes, it is possible that the hyper-accumulation of thymidine in HopQ1 lines is a secondary effect. It is also possible that HopQ1 does not directly target nucleosides, but affects nucleoside metabolism indirectly through other host targets. Taken together, our nucleoside profiling data indicate that HopQ1 affects host metabolism.

### HopQ1 cannot Cleave or Bind Standard Nucleosides *in vitro*

**Figure 6:**
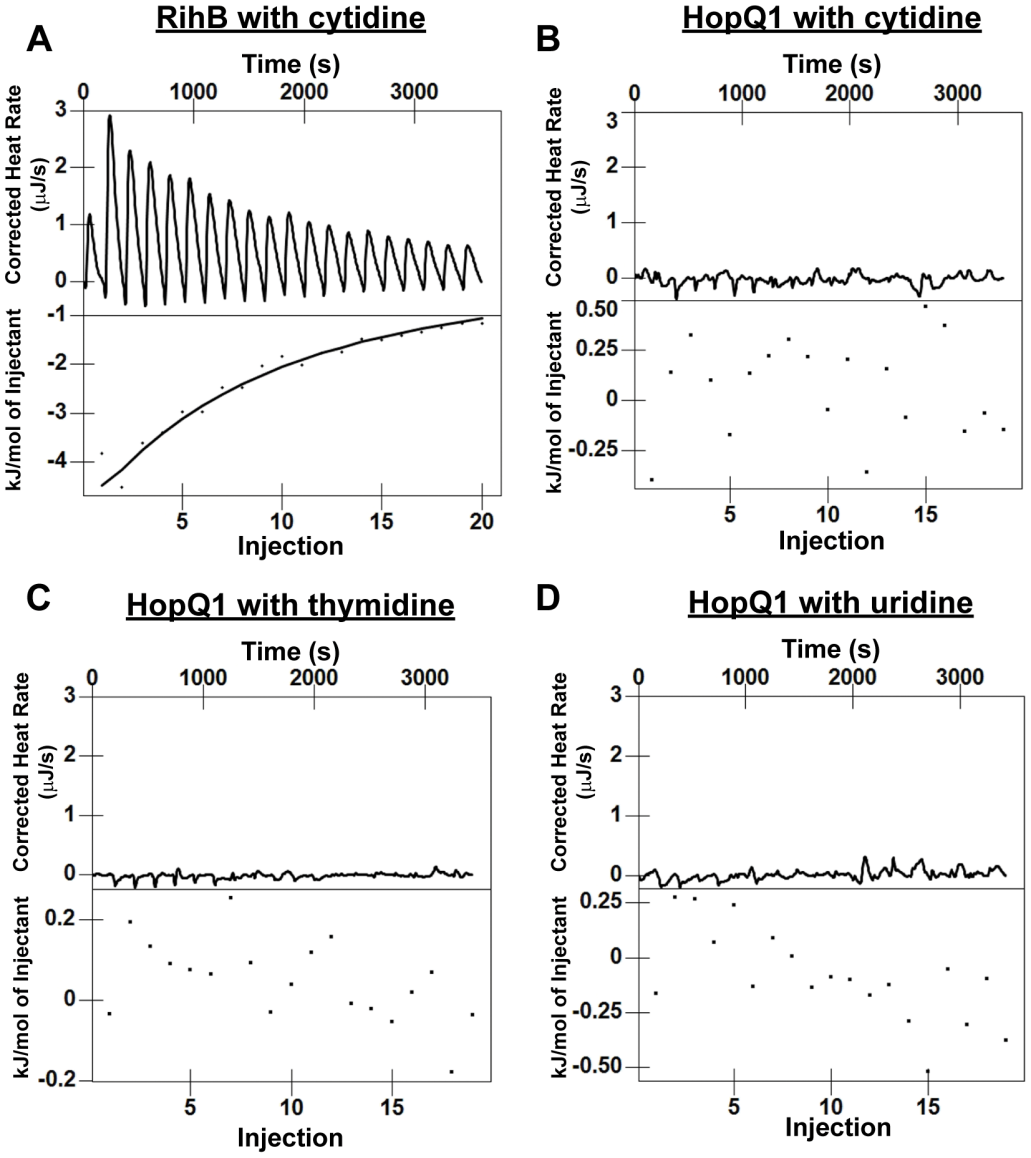
Recombinant HopQ1 does not bind the nucleosides cytidine, thymidine, or uridine using isothermal titration calorimetry. HopQ1∶6XHistidine was expressed and purified from HiFive lepidopteran cells. The uridine-cytidine preferring nucleoside hydrolase RihB was expressed and purified as a maltose-binding protein (MBP) fusion from *E. coli.* Isothermal titration calorimetry (ITC) profiles corresponding to the binding of RihB or HopQ1 to selected nucleosides are shown. (A) 1.6µM of MBP-RihB with 20 mM cytidine (B) 4.9µM of HopQ1-His with 20 mM cytidine (C) 1µM of HopQ1-His with 20 mM thymidine, and (D) 4.9µM of HopQ1 with 20 mM uridine. Upper panels show the power differential recorded over time. Lower panels show the calculation of integrated heat of binding obtained from the raw data after multiple nucleoside injections. The solid line in the lower panel of (A) represents the best curve fit to the experimental data, using an independent binding model from NanoAnalyze.

We have been able to purify recombinant HopQ1 expressed as a maltose binding protein (MBP:HopQ1) fusion from *E. coli* and Histidine fusions (HopQ1∶6XHis) after expression in High Five lepidopteran (insect) cells (Fig S4). Nucleoside hydrolase enzymatic activity was examined by using all standard nucleosides as substrates (cytidine, uridine, inosine, thymidine, adenosine, and guanosine). As expected, the purified pyrimidine-specific nucleoside hydrolase MBP:RihB showed high enzymatic activity for cytidine and uridine, and intermediate activity for inosine (data not shown), consistent with the results from Petersen and Moller [25]. However, we did not detect any nucleoside hydrolase activity among all the standard nucleosides by using either MBP:HopQ1 and HopQ1∶6XHis (data not shown). We then examined if HopQ1 can bind nucleosides. The binding interaction between HopQ1∶6XHis and nucleosides were examined by isothermal titration calorimetry. Recombinant protein MBP:RihB purified from *E. coli* was used as positive control. MBP-RihB exhibited binding to cytidine at a concentration of 20 mM (Fig. 6), consistent with its enzymatic preference for cytidine. However, HopQ1∶6XHis protein did not bind to cytidine, thymidine, and uridine (Fig. 6). Together, these results suggest that HopQ1 likely hydrolyzes alternative ribose-containing substrate(s).

## Discussion

Bacterial pathogens manipulate host defense responses by delivering effectors into host cells during infection. Multiple effectors have been shown to target host immune receptors and downstream signaling proteins [3]. However, the role of effectors in modulating host metabolism by targeting small molecules remains largely unexplored. Previous experiments have demonstrated that *P. syringae* is able to rapidly and robustly induce host metabolic changes during infection [10]. These metabolic changes occur within 8 hours post-inoculation, a time at which multiple effectors are delivered into host cells [10]. *P. syringae* mutants that cannot deliver type III effectors are unable to induce changes in host metabolism, indicating that bacterial effectors may play a role in modulating metabolism for pathogen benefit [10]. Here, we provide evidence that *Pto* HopQ1 possesses some similarity to nucleoside hydrolases, and conserved putative NH catalytic residues are required for this effector’s virulence-promoting activities in tomato and Arabidopsis.

Nucleoside hydrolases cleave nucleosides generating a ribose and respective nucleotide base. Degradation of nucleosides into simple metabolites enables the recycling of phosphate, nitrogen, and carbon metabolic pools. Mobilization of nitrogen occurs during pathogen infection and in compatible interactions, can act to benefit the pathogen and deprive the plant of nutrients [26]. With respect to purines, xanthosine metabolism leads to the production of the ureides allantoin and allantoate [27]. Allantoin and allantoate can act as ROS scavengers, attenuate stress responses, and accumulate under senescence [28], which may also have important implications in plant-pathogen interactions. Consistent with this finding, knockouts of the Arabidopsis nucleoside hydrolase NSH1, which can hydrolyze both uridine and xanthosine, exhibit accelerated senescence in the dark [29]. Adenosine hydrolysis can also significantly impact on cellular energy status by disrupting ATP homeostasis. Due to their importance in multiple facets of plant metabolism, nucleoside salvage/degradation and related pathways may be attractive pathogen targets.

Despite multiple attempts, we were unable to detect enzymatic activity or nucleoside binding using recombinant HopQ1 purified from *E. coli* or lepidopteran cells or HopQ1 protein purified from transgenic plants. It is possible that recombinant HopQ1 is not properly folded or active in our buffer conditions, but HopQ1 expressed from *E. coli* migrates normally by gel filtration chromatography, indicating that HopQ1 is not misfolded (data not shown). In contrast, the characterized NH RihB from *E. coli* exhibited strong NH activity and NH binding using ITC (Fig. 6). We also tried adding crude plant extract to NH enzyme assays as well as a variety of potential co-factors in the event HopQ1 may require additional plant co-factors or proteins for activity, to no avail. Nevertheless, HopQ1’s central region possesses significant similarity to characterized NHs and mutation of conserved catalytic residues affects HopQ1’s virulence promoting activities *in planta*. It is important to note that HopQ1 effectors contain a variation from the classic NH N-terminal hallmark DXDXXXDD. Thus, we hypothesize that HopQ1 possesses an NH fold, but acts to hydrolyze or bind alternative plant-specific ribose containing substrates.

Investigating HopQ1 function in both *Nicotiana* and Arabidopsis revealed that this effector is a modular protein whose NH-like domain is sufficient for promoting bacterial virulence. Although HopQ1 can promote bacterial virulence, we did not detect compromised ROS burst or MAP kinase activation after flg22 treatment in transgenic plants expressing HopQ1 (data not shown). Potentially HopQ1 could be altering host metabolism and enhancing pathogen virulence in a novel way that does not interfere with immune signaling networks *per se.* The C-terminus of HopQ1, which is widely conserved across other homologs, is not required for virulence promotion (Figs 3, 4, 5), but is required for recognition in *Nicotiana* (Figs 2 and S2). Our mutational analyses and short deletions within HopQ1’s NH domain suggest that NH activity *per se* is not required for recognition. However, large deletions within the NH domain lose their ability to elicit cell death. Thus, the NH fold may be recognized. Alternatively, an intact NH domain may be required for proper folding of the effector or C-terminus. Future investigations on the function of HopQ1’s C-terminus, elucidating HopQ1’s true substrate(s) in plants, and investigating HopQ1-mediated alterations in diverse host metabolites will shed light onto how host metabolism can be specifically manipulated for pathogen benefit.

## Materials and Methods

### Structural Modeling

A structural model for the HopQ1 protein was generated using the *E. coli* IU-NH RihB as a template (PDB ID = IQ8F). The MODELLER soft- ware package [19] was used to generate a protein structural model of HopQ1 with 10 optimizations. Graphical images were produced with the UCSF Chimera package from the Resource for Biocomputing, Visualization, and Informatics at the University of California, San Francisco [30].

### HopQ1 Plasmids and Constructs

The HopQ1 open reading frame was PCR amplified from *Pto* DC3000 and cloned into pENTR/D-TOPO. Mutants for single amino acid residues were generated by PCR mutagenesis. Internal deletion constructs were generated by overlap PCR of two independent amplicons. Truncations were directly amplified from a full-length clone. Resulting PCR products were cloned into pENTR/D-TOPO (Invitrogen) and then sub-cloned into respective gateway vectors. All primers used for PCR and cloning are listed in Table S1.

For inducible expression in *Arabidopsis,* HopQ1 clones were introduced into the pTA7001 binary vector under the control of the Gal4-VP16-glucocorticoid receptor-induced promoter [31]. pTA7001 was modified to be gateway compatible with a C-terminal 3XFLAG tag. The 3XFLAG amino acid sequence (DYKDHDGDYKDHDIDYKDDDDK) was codon optimized for expression in plants and cloned into pCR2.1 (Invitrogen) as a *Sal*I/*Not*I fragment. The coding sequence for the Gateway recombination cassette (containing the ccdB gene, CAT chloramphenicol resistance gene, and attR recombination sites) was amplified and cloned in front of 3XFLAG as an *Xho*I/*Sal*I fragment. The gateway cassette 3XFLAG fusion was then cut out of pCR2.1 and ligated into pTA7001 as a *Xho*I/*Spe*I fragment to generate pTA7001/des/3XFLAG.

For transient expression experiments in *Nicotiana*, HopQ1 clones were introduced into pEarleyGate103 [32] for in-frame fusions to GFP. For expression in *Pto* DC3000, HopQ1 and related clones were introduced into the broad host range vector pBBR1-MCS5 with a C-terminal 3xFLAG tag under the control of the AvrB promoter [22]. For expression in *P. syringae* pv. *tabaci*, HopQ1 and related clones were introduced into the broad host range vector pBAV226 [33].

### LC-MS Metabolic Profiling

Quantification of cellular levels in was conducted in three-week-old *Arabidopsis* seedlings expressing GFP or HopQ1∶3xFLAG grown on MS media supplemented with 30µM dexamethasone (Dex). One hundred mg of seedling samples were extracted with 300µL of methanol/water mixture 3∶1 v.v. Ten µL was injected onto the column. The experiments were conducted at University of California, Davis metabolomics core facility. Individual nucleoside peak areas (cytidine, uridine, inosine, thymidine, guanosine, adenosine and guanosine) were visually inspected to ensure that peaks were properly selected and appropriate bounds were used.

Chromatographic separation was performed using a Waters Acquity UPLC (Milford, MA, USA).

Waters UPLC BEH C18 column (150 mm × 2.1 mm i.d., 1.7 µm particle size) was used for separations. The mobile phases were 0.1% formic acid in water (A) and acetonitrile (B). Column temperature was set to 60°C. The injection volume was 10 ml with a partial loop method of injection. A weak wash was performed with water as the solvent. A strong wash was performed with acetonitrile as the solvent. Metabolites were separated with a linear gradient (300 µl/min flow rate) from 0% to 15% (B) over 5 min followed by a 1 min hold at 100% (B) (at a 400 µl/min flow rate). The mobile phase was returned to 0% (B) at 7 min and the column was re-equilibrated for 3 min prior to the next run. MS/MS detection was carried out using API 4000 Q-Trap hybrid triple quadrupole linear ion trap mass spectrometer (Applied Biosystems/MDS SCIEX, Foster City, CA, USA) operated in positive ion MRM mode. Automated peak detection and integration were used to determine peak area and signal to noise ratio.

### Expression and Purification of Recombinant Proteins

RihB cDNA was PCR-amplified from the *E.coli* (DH5α) genome. RihB and HopQ1 PCR products with *Bam*HI and *Xho*I enzyme cutting sites at their N- and C-termini, respectively, were sequenced and sub-cloned into the corresponding sites of the pMAL-c4X vector (Invitrogen) to generate N-terminally tagged maltose binding protein (MBP) fusions. MBP-tagged constructs were transformed into *E. coli* Rosetta (DE3) cells (Novagen). Protein expression was induced with 0.5 mM isopropyl-1-thio-β-d-galactopyranoside when the *A*_600_ reached 0.4–0.6. Cultures were then grown for 4 h at 28°C, and the bacterial pellets were harvested by centrifugation. MBP tagged proteins were purified by using amylose resin (New England Biolabs) according to the manufacturer’s protocol.

For expression HopQ1 in lepidopteran cells, the coding sequence of HopQ1 was cloned into the pFastBac/CT-TOPO vector (Invitrogen) and transformed into DH10Bac *E. coli* cells (Invitrogen) according to the manufacturer’s manual. High molecular weight recombinant bacmid DNA was isolated from DH10Bac cells and then transfected into Sf9 insect cells (Novagen) to generate recombinant baculovirus for infecting high five insect cells. Soluble HopQ1∶6XHis protein was purified from infected high five cells by Ni-NTA agarose (QIAGEN) and subjected to NH enzymatic assays according to [34].

### Isothermal Titration Calorimetry

The interaction of RihB and HopQ1 with different nucleosides was investigated using a nano isothermal titration calorimeter (Nano ITC) (TA instruments). Purified recombinant proteins and nucleoside solutions were prepared in 50 mM sodium phosphate buffer pH 7.2. Twenty consecutive injections of 2.5µl of 20 mM nucleosides were added with a paddle stirrer-syringe into the calorimeter cell filled with 250µl MBP:RihB or HopQ1∶6XHis protein solution. Injections were made at intervals of 2 minutes for all titrations. The corrected data were analyzed and best curves were generated by using an independent binding model from NanoAnalyze (TA instruments).

### Plant Materials and Growth Conditions

All HopQ1 binary vectors were electroporated into *Agrobacterium tumefaciens* strain *GV3101*. Transgenic Arabidopsis lines (ecotype Columbia-0) were generated by floral dip [35] and selected on ½ MS medium supplemented with 50 µg/ml hygromycin. All experiments were conducted on T3 homozygous lines. All experiments were repeated at least three times, with a minimum of three biological replicates per time point. Inducible lines and controls were sprayed with 30 µM Dex with 0.02% silwett 24 h before pathogen treatment.

### Bacterial Strains and Growth Curves

Inoculations and bacterial growth curves in Arabidopsis were conducted by syringe infiltration as previously described using *Pseudomonas syringae* pv. *tomato* (*Pto*) DC3000 [36]. Inoculations of *P. syringae* pv. *tabaci* on *N. benthamiana* were performed as previously described [15]. Blight symptoms were observed 72 h post-inoculation.

### Immunoblotting

SDS-PAGE and subsequent immunoblots were performed according to standard procedures. Anti-FLAG immunoblots were performed at a concentration of 1∶1,000 (F1804, Sigma). GFP immunoblots were performed at a concentration of 1∶5,000 (ab290, Abcam). Secondary goat anti-rabbit or goat anti-mouse IgG-HRP conjugate (Biorad) was used at a concentration of 1∶3,000 for detection via enhanced chemiluminescence (Pierce). Bacterial effector expression and secretion was performed as previously described after growth in hrp-inducing minimal media [37].

### Transient Expression in *Nicotiana*

*Agrobacterium*-mediated transient expression was performed as described previously [38]. For HR assays, HopQ1 and related clones were electroporated into *A. tumefaciens* strain GV3101. GFP in the binary vector pMDC43 was used as a control. *Agrobacteria* were infiltrated into tobacco leaves at an OD_600_ = 0.4. HR phenotypes were recorded 72 h post-inoculation.

## Supporting Information

Figure S1 **HopQ1 is widely conserved across phytopathogenic bacteria.** HopQ1 from *P. syringae pv. tomato* DC3000 (accession number NP-790716) aligned with homologs from *P. syringae pv. phaseolicola 1448A* (YP_272139), XopQ from *Xanthomonas gardneri* ATCC 19865 (ZP_08182005), XopQ from *X. campestris pv. campestris* str. 8004 (YP_244241), Ripb from *Ralstonia solanacearum* CMR15 (CBJ39448), and *Acidovorax avenae* subsp. *avenae* ATCC 19860 (YP 004236009). Amino acid numbers correspond to the HopQ1 protein sequence. Only one homolog is included from each bacterial species. Identical amino acids are shaded black; similar residues are shaded light grey. Conserved putative NH active site residues are indicated by an asterisk above such a position.
(TIF)

Figure S2 **HopQ1’s nucleoside hydrolase-like domain is not required for recognition in** ***N. tabacum*** **when expressed from** ***P. syringae pv. tabaci.*** *P. syringae* pv. *tabaci* expressing HopQ1(65–477) and HopQ1(1–390) deletions recover blight symptoms on *N. benthamiana*. Constructs were expressed in *P. syringae pv. tabaci* from the broad host range vector pBAV226, infiltrated into *N. benthamiana* at a concentration of 5x10^5^ cfu/cm2, and blight symptoms photographed 72 h post-inoculation.
(TIF)

Figure S3 **Arabidopsis plants expressing HopQ1 possess altered nucleoside levels.** (A-F) Quantification of cellular nucleoside (uridine, cytidine, thymidine, inosine, adenosine and guanosine) levels in three-week-old *Arabidopsis* seedlings grown on MS media supplemented with 30µM dexamethasone (Dex). HopQ1 expression in *Arabidopsis* is under the control of a Dex-inducible promoter. T3 homozygous plants were used for metabolic profiling and two independent experiments were conducted. A buffer control was set as the blank. Statistical differences were detected by Fisher’s LSD, alpha  = 0.05. Error bars represent means (n  = 6) ± SD.
(TIF)

Figure S4 **Recombinant protein expression and purification** SDS-PAGE of purified maltose binding protein (MBP) fusions MBP:HopQ1 and MBP:RihB from *E.coli*, and HopQ1∶6XHis from lepidopteran cells. HopQ1∶6XHis is cleaved on its N-terminus at two sites in lepidopteran cells. All three bands of HopQ1∶6xHis are detectable by anti-His immunoblot analyses. Asterisks indicate full-length protein.
(TIF)

Table S1 **Primers used for cloning.**
(DOCX)
