# Supplementary figures and images for: The HopQ1 Effector’s Nucleoside Hydrolase-Like Domain Is Required for Bacterial Virulence in Arabidopsis and Tomato, but Not Host Recognition in Tobacco
Source: PLoS One. 2013 Mar 26;8(3):e59684. doi: 10.1371/journal.pone.0059684 (PMC3608555; doi:10.1371/journal.pone.0059684)

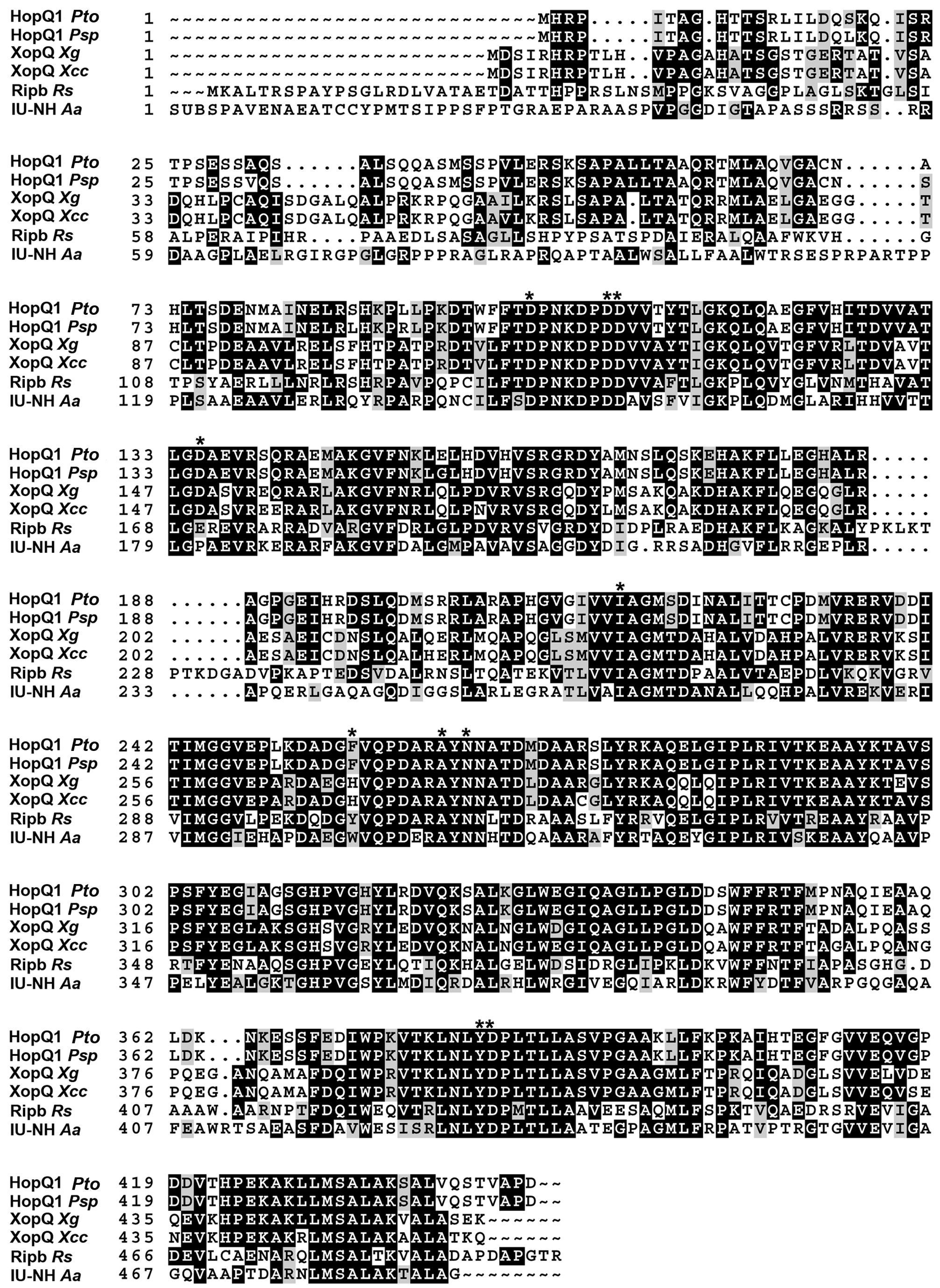

Supplement: Figure S1 — HopQ1 is widely conserved across phytopathogenic bacteria. HopQ1 from P. syringae pv. tomato DC3000 (accession number NP-790716) aligned with homologs from P. syringae pv. phaseolicola 1448A (YP_272139), XopQ from Xanthomonas gardneri ATCC 19865 (ZP_08182005), XopQ from X. campestris pv. campestris str. 8004 (YP_244241), Ripb from Ralstonia solanacearum CMR15 (CBJ39448), and Acidovorax avenae subsp. avenae ATCC 19860 (YP 004236009). Amino acid numbers correspond to the HopQ1 protein sequence. Only one homolog is included from each bacterial species. Identical amino acids are shaded black; similar residues are shaded light grey. Conserved putative NH active site residues are indicated by an asterisk above such a position. (TIF) [file pone.0059684.s001.tif]

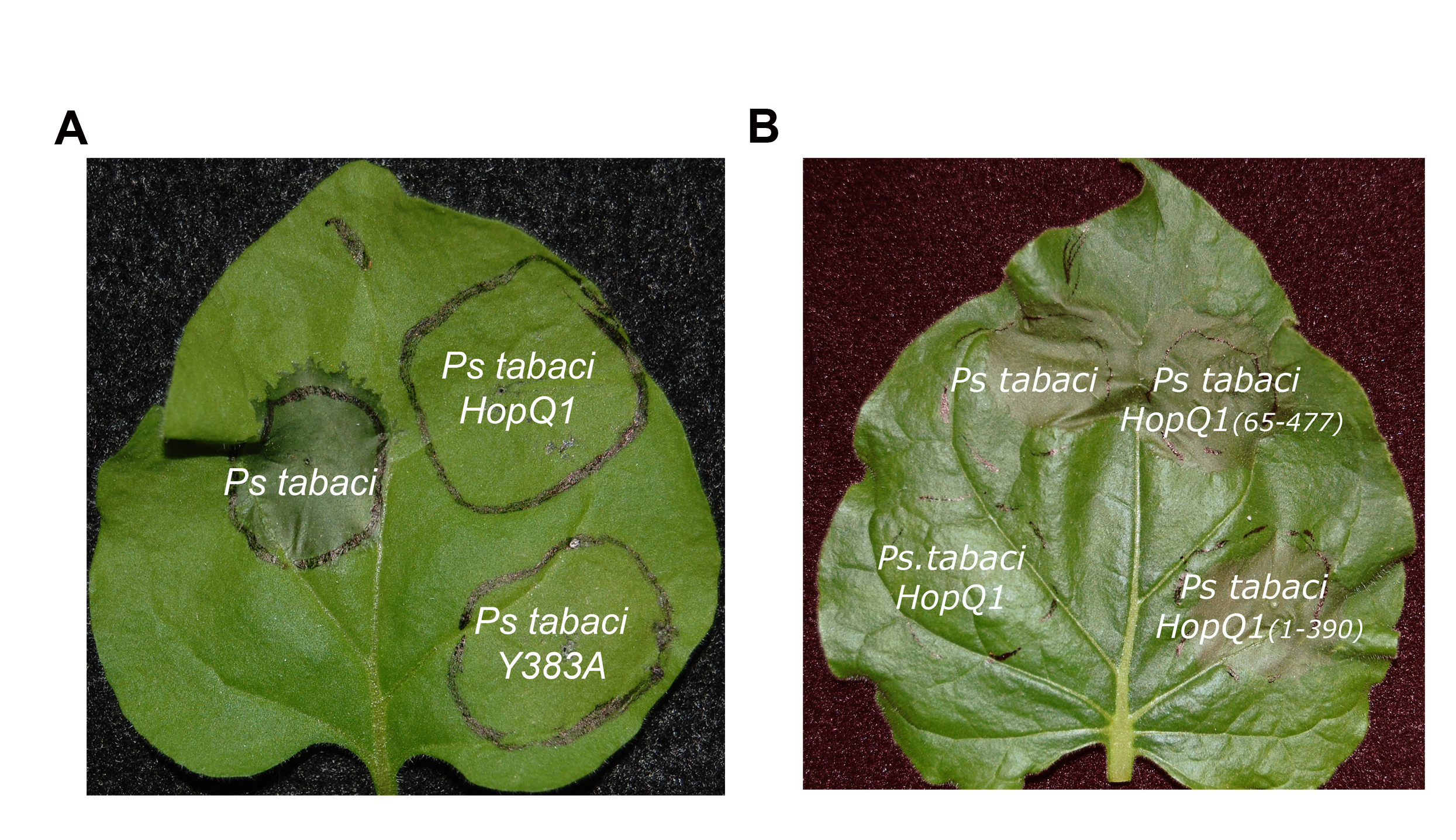

Supplement: Figure S2 — HopQ1’s nucleoside hydrolase-like domain is not required for recognition in N. tabacum when expressed from P. syringae pv. tabaci. P. syringae pv. tabaci expressing HopQ1(65–477) and HopQ1(1–390) deletions recover blight symptoms on N. benthamiana. Constructs were expressed in P. syringae pv. tabaci from the broad host range vector pBAV226, infiltrated into N. benthamiana at a concentration of 5x10^5^ cfu/cm2, and blight symptoms photographed 72 h post-inoculation. (TIF) [file pone.0059684.s002.tif]

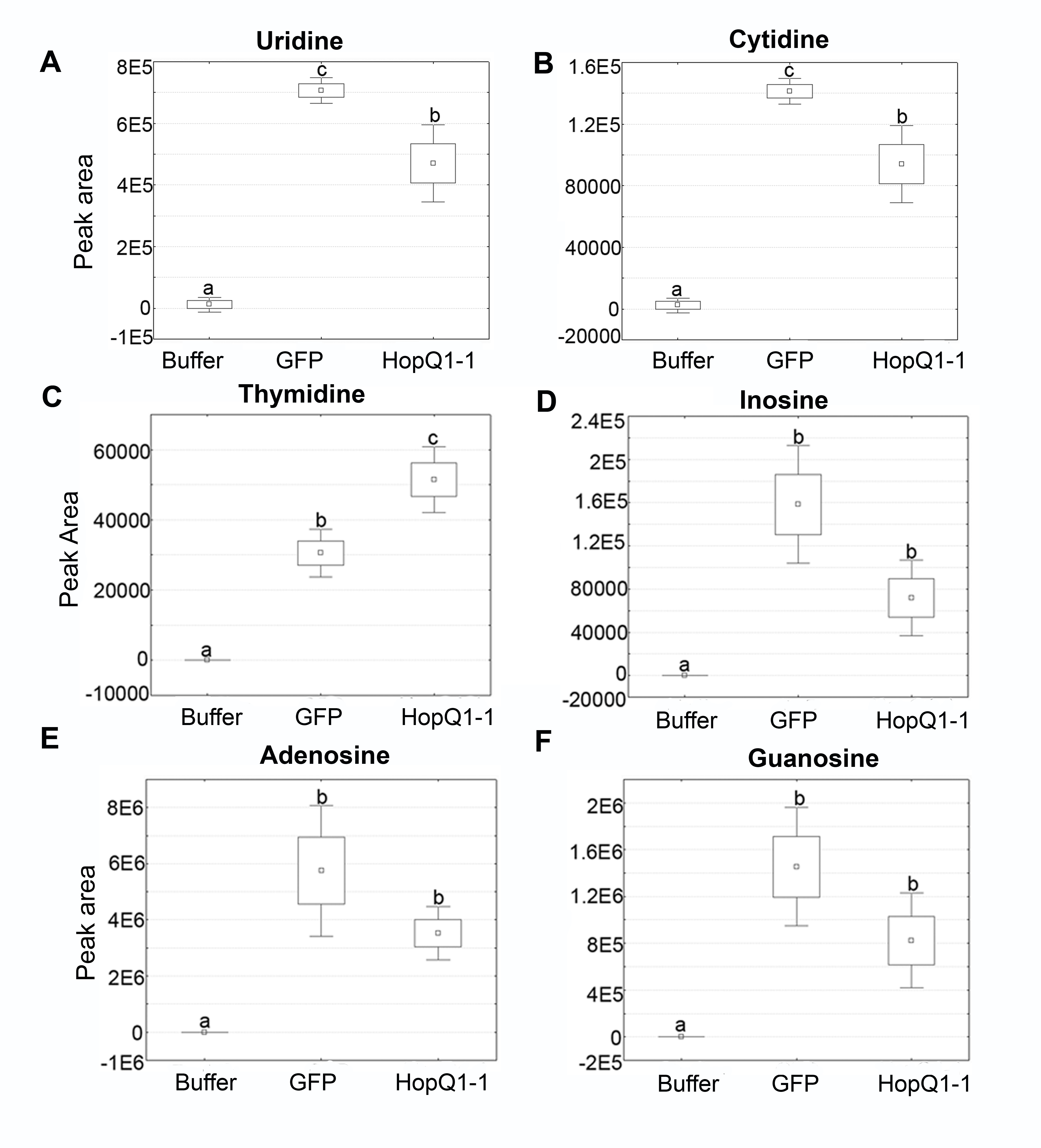

Supplement: Figure S3 — Arabidopsis plants expressing HopQ1 possess altered nucleoside levels. (A-F) Quantification of cellular nucleoside (uridine, cytidine, thymidine, inosine, adenosine and guanosine) levels in three-week-old Arabidopsis seedlings grown on MS media supplemented with 30µM dexamethasone (Dex). HopQ1 expression in Arabidopsis is under the control of a Dex-inducible promoter. T3 homozygous plants were used for metabolic profiling and two independent experiments were conducted. A buffer control was set as the blank. Statistical differences were detected by Fisher’s LSD, alpha = 0.05. Error bars represent means (n = 6) ± SD. (TIF) [file pone.0059684.s003.tif]

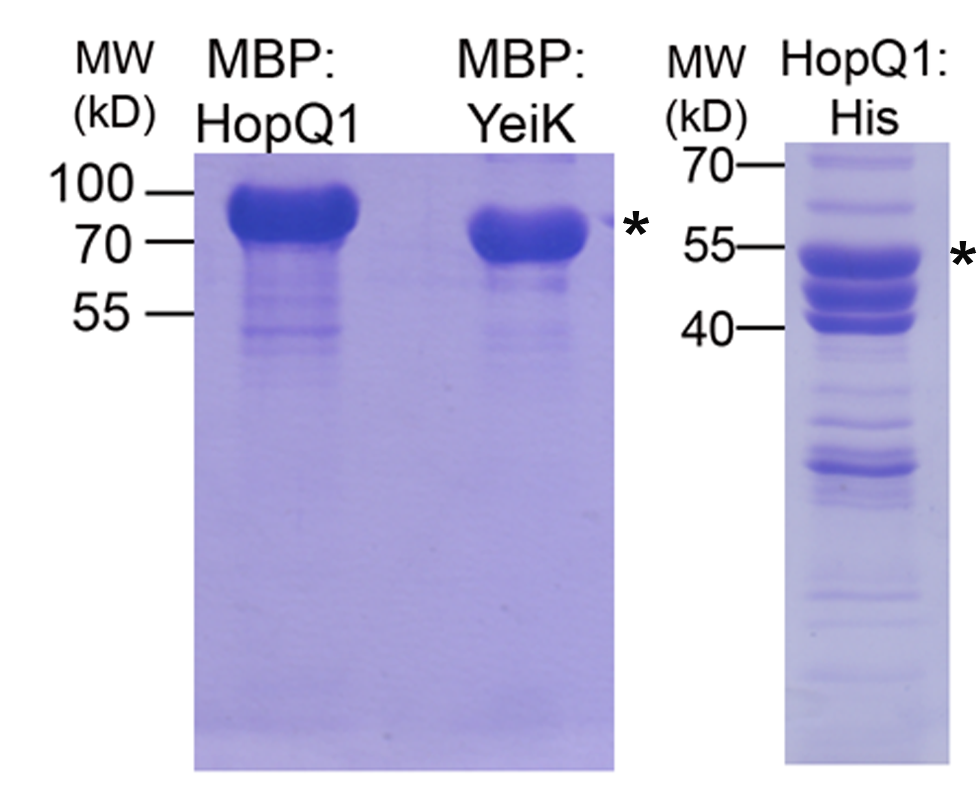

Supplement: Figure S4 — Recombinant protein expression and purification SDS-PAGE of purified maltose binding protein (MBP) fusions MBP:HopQ1 and MBP:RihB from E.coli, and HopQ1∶6XHis from lepidopteran cells. HopQ1∶6XHis is cleaved on its N-terminus at two sites in lepidopteran cells. All three bands of HopQ1∶6xHis are detectable by anti-His immunoblot analyses. Asterisks indicate full-length protein. (TIF) [file pone.0059684.s004.tif]
