# Supplementary material for: The HopQ1 Effector’s Nucleoside Hydrolase-Like Domain Is Required for Bacterial Virulence in Arabidopsis and Tomato, but Not Host Recognition in Tobacco
Source: PLoS One. 2013 Mar 26;8(3):e59684. doi: 10.1371/journal.pone.0059684 (PMC3608555; doi:10.1371/journal.pone.0059684)
Supplement: Table S1 — Primers used for cloning. (DOCX) [file pone.0059684.s005.docx]

**Table S1 Primers used for cloning**

| Primer Name | Primer Sequence5’-3’ |
| --- | --- |
| HopQ1-F | CACCATGCATCGTCCTATCACCGCA |
| HopQ1-R | ATCTGGGGCTACCGTCGACTG |
| HopQ1(D108A)-F | AGGACCCAGATGCTGTCGTG |
| HopQ1(D108A)-R | CACGACAGCATCTGGGTCCT |
| HopQ1(Y383A)-F | GACGAAGCTAAACCTGGCTGATCCTCTGACATTAC |
| HopQ1(Y383A)-R | GATATGTCAGAGGATCAGCCAGGTTTAGCTTCGTC |
| HopQ1(D384A)-F | AACCTGTATGCTCCTCTGAC |
| HopQ1(D384A)-R | GTCAGAGGAGCATACAGGTT |
| HopQ1_∆101-110_-F | CCCAAGGTGTAGGTAGTGAAA |
| HopQ1_∆101-110_-R | GGTGGTTTTTCACTACCTACA |
| HopQ1_∆263-265_-F | CCTGATGCACGCAATGCGACCGACATGGACGC |
| HopQ1_∆263-265_-R | GTCGGTCGCATTGCGTGCATCAGGCTGTACAA |
| HopQ1_(65-477_)-F | CACCATGGCACAAGTGGGAGCCTGT |
| HopQ1_(1-64)-_R | AAGCATCGTGCGCTGTGC |
| HopQ1_(1-390)_-R | GGCCAGTAATGTCAGAGGAT |
| HopQ1_(390-477)_-F | CACCATGGCACAACTGGATAAAAATAAA |
| HopQ1_(1-430)_-R | CATCAATAACTTTGCTTTCT |
| HopQ1_(90-477)_-F | CACCATGCTTTTACCTAAGGATACGTG |
| HopQ-1-1_(1-100)_-R | ATCATATAGGTTAGTGAAAAACCACGTATCCT |
| HopQ1_(1-380)_-F | TGGTTTTTCACTAACCTGTATGATCCTCTGAC |
| HopQ1_∆420-430_-F | GGTCCAGATGATATGTCCGCTTTAGCCAAATC |
| HopQ1_∆420-430_-R | TAAAGCGGACATATCATCTGGACCTACTTGCT |
| SalI 3XFLAG-F | CGTCGTGTCGACGATTATAAGGATCATGATGGAGATTATAAGG  ATCATGATATTGATTAT |
| 3X FLAG SpeI NotI-R | CCATGCGCGGCCGCACTAGTTTACTTATCATCATCATC  CTTATAATCAATATCATGATCC |
| XhoI Gateway-F | GCACGGCTCGAGACAAGTTTGTACAAAAAAGC |
| Gateway XhoI-R | CCTGCCTCGAGCACCACTTTGTACAAGAAAGC |
